# The effect of different combination therapies on oxidative stress markers in HIV infected patients in cameroon

**DOI:** 10.1186/1742-6405-3-19

**Published:** 2006-07-22

**Authors:** Judith L Ngondi, Julius Oben, David Musoro Forkah, Lucten Honore Etame, Dora Mbanya

**Affiliations:** 1 Nutrition, HIV and Health Research Unit, Department of Biochemistry, University of Yaounde I, Cameroon; 2 Faculty of Medicine and Biomedical Sciences, University of Yaounde I, Cameroon

## Abstract

The study assessed the effect of some highly active antiretroviral therapies (HAART), used in the management of HIV/AIDS in Cameroon, on oxidative stress markers such as malondialdehyde (as TBARs), albumin, protein carbonyl content and protein sulfhydryls groups. 85 HIV positive patients (34.8 ± 9.3 years) were on three different highly active antiretroviral therapies (HAART patients). 65 HIV positive patients (32.2 ± 10.9 years) on no treatment (Pre-HAART patients), and 90 non-HIV infected patients (32.6 ± 9.3 years), were the control groups. Plasma TBARs as well as carbonyl levels were significantly higher in HIV patients on HAART compared to pre-HAART patients or non-HIV infected controls. On the other hand, the protein sulfhydryl group content was not different for patients on HAART compared to pre-HAART patients, but both were significantly lower than non-HIV infected controls (P < 0.0001, 0.001). The combination treatment Therapy I [stavudin (80 mg) + Lamivudin (600 mg) + Nevirapin + (400 mg) zidovudin (600 mg)] brought about a significant (p < 0.05) reduction in the plasma concentration of protein sulfhydrl groups as well as TBARs compared to Therapy II [stavudin (80 mg) + Lamivudin (300 mg) + nevirapin (400 mg)] or with combination Therapy III of [zidovudine (600 mg) + lamivudin(300 mg) with efavirenz (600 mg)] (P < 0.05). The content of the antioxidant, Vitamin C was lower in the plasma of patients on Therapy I compared to those on Therapy II (P < 0.01) and Therapy III (P < 0.001).

HIV infection therefore increases the oxidative stress process, while antiretroviral combination therapy increased protein oxidation as well as the level of oxidative stress already present in HIV infection.

## Introduction

The use of antiretroviral therapies (ART) is recommended worldwide for the management of HIV/AIDS. Different types of ART or combination therapies are available, and the prescription and use of a particular therapy depends on tolerabity, the cost, and the therapeutic objectives. The initiation of therapy is dependent on diagnosis as well as serological parameters, with therapy generally started when the CD4 count is less than 200 lymphocytes/mm^3^. WHO currently recommends first-line therapy with two nucleoside reverse transcriptase inhibitors (NRTIs) and one non-nucleoside reverse transcriptase inhibitors (NNRTI) [1]. Because of the low income of hour population, the Cameroon Ministry of public Health is continuously looking for generic drugs which are generally cheaper. A combination of nevirapin, stavudin, and Lamivudin or lamivudin with zidovudin is frequently prescribed. Different information and refresher seminars are organised to educate medical practitioners and health workers on the use and prescription of these therapies, which still varies from one health facility to another. Some of the treatment options used may result in biochemical and physiological changes e.g. oxidative damage resulting from the presence of free radicals or the absence of antioxidants.

Free radicals are compounds possessing an unpaired electron, which renders them highly reactive and capable of causing oxidative damage to all the major macro-molecules in cells, including lipids, proteins and nucleic acids. A major family of free radicals is the reactive oxygen species, derived metabolically from molecular oxygen via super oxide anions. Oxidative attack on proteins results in the formation of protein carbonyls [2], often with the loss of functionality of the parent protein. Polyunsaturated fatty acids, which are major components of cell membranes, can also undergo free radical attack, producing lipid peroxidation products like malondialdehyde (MDA) and 4-hydroxynonenal. Under normal circumstances, the body is protected from such damage by a careful balance between pro-oxidants and antioxidants. In extra vascular spaces, the sulphydryls group of plasma proteins, including plasma albumin serve as antioxidants [3], with enzymes and scavenging chemicals such as Vitamin C and vitamin E also having antioxidant activities. Certain foods are good sources of antioxidants, hence the importance of their inclusion in the treatment of illnesses accompanied by oxidative stress. Although there are several known side effects of HIV/AIDS medications, like lipodystrophy, an increase in the risk of cardiovascular disease, including heart attacks and stroke etc., there is not sufficient evidence to substantiate the role of free radicals in mediated oxidative injury in HIV/AIDS infection. This study evaluated the oxidant and antioxidant status of HIV positive patients in Cameroon.

## Methods

### Patients

Patients were eligible if they had confirmed HIV infection, were older than 18 years, and had not taken antiretroviral drugs before (pre-HAART patients) or being on triple drugs therapy for least 3 to 6 months. HIV positive out patients (150) were recruited from the Yaounde Hospital. Patients were classified according to their sex, immune status such as the CD4 + count (lymphocytes/mm^3^) and viral load measurements. 65 positive patients were not on any form of antiviral therapy, while 85 were on one of the different therapies outlined below. These individuals showed no serological evidence for HBV and or HCV-infection. The control group comprised of 90 randomly selected age-matched healthy subjects who showed no serological evidence for HIV and/or HCV-infection and no abnormal laboratory finding (blood count, ASAT, ALAT levels were all within the normal range).

All subjects gave their informed consent before participating in the study, and the local ethics committee approved the project.

### Different Highly active anti retroviral Therapies (HAART) used

Patients on antiviral therapy followed one of the following daily therapies:

Therapy I (n = 40): Generic fixed-dose combination of stavudin + lamivudin + nevirapin associated with a generic fixed-dose combination of zidovudin +Lamivudin;

Therapy II (n = 20): Generic fixed-dose combination of stavudin+ lamivudin + nevirapin

Therapy III (25): Generic fixed dose combination of zidovudin + lamivudin associated with efavirenz.

### Sample collection and treatment

Over night (12 hour) fasting venous blood (10 ml) was collected from all subjects and divided between a heparinized tube and a tube containing Ethylene Diamine Tetra Acetate (EDTA). After centrifugation at 3000 g for 10 minutes at 4°C, a sample of the heparinized plasma was set aside for the analysis of protein sulphydryls and protein carbonyls. Samples of plasma containing EDTA were also set aside for the measurement of Vitamin C and MDA. All samples were stored at -80 °C and analysed within 2 weeks.

### Biochemical analyses in plasma

Total protein was determined using a commercially available Biuret method [4]. Lipid peroxidation was measured by the thiobarbituric acid method described in [5], and results expressed as μmol/l. Protein carbonyl, albumin and protein sulphydryls concentrations were measured spectrophotometrically were determined using the methods of [6-8], Plasma Vitamin C concentration was determined by the method of Roe and Kuether [9].

### Statistical analysis

Values are expressed as mean ± Standard error of mean (SEM). Data was analysed using the SPSS package, and normality of distribution of the data assessed using the normal plot method. The variables were normally distributed and comparisons made using ANOVA test. Differences between groups were assessed using the Bonferonni procedure.Multiple linear regression analysis was used to assess the influence of influence of BMI, age, sex, CD4 count and Viral load on oxidative stress markers in HAART patients. A *p *value of = 0.05 was considered significant

## Results

### Immunological characteristics

The CD4 count was significantly (p < 0.001) lower in the HIV positive subjects on HAART compared to pre-HAART patients (Table 1). The viral loads were also found to be significantly higher for patients on HAART.

**Table 1:** Characteristics of HIV positive patients on antiviral therapy, without therapy and controls

	**HIV**^-^	**pre-HAART**	**HAART**
**n**	90	65	85
**Sex (M/F)**	20/70	27/38	30/55
**Age (years)**	34 ± 9.15	33.71 ± 8.56	36.8 ± 8.91
**BMI (kg/m^2^)**	25.31 ± 2.44	25.01 ± 3.66	25.19 ± 4.70
**CD4 **(lymphocytes/mm^3^)	/	496 ± 163	266 ± 137^a^
**Viral load**	/	26754 ± 8347	111136,33 ± 25281^a^

Significant differences (^a^P < 0.0001) are by comparison with the HIV positive patients without therapy (pre-HAART)

### Plasma analysis

Measures of lipids and protein oxidation showed a significantly elevated levels of TBARs (P < 0.0001, 0.0001) and lower levels of carbonyl (P < 0.0001, 0.0001) in HIV patients on antiviral therapy compared to those not following therapy or non HIV infected controls (Table 2). On the other hand, plasma protein sulfhydryls group (Table 3) was not different for patients on HAART compared to pre-HAART patients, but both were significantly lower than non HIV infected controls (P < 0.0001, 0.001). Plasma albumin levels were lower in HIV positive patients, no difference were found in the level of vitamin C (Table 3). Patients on Therapies I, II and III had lower concentrations of carbonyls (p < 0.001) and elevated concentrations of TBARs (p < 0.001) compare to pre-HAART patients (Table 4). Plasma concentrations of sulfhydryls group of patients on therapy II (p = 0.01) was lower compare to pre-HAART patients but their albumin level (p < 0.01) was higher compare to pre-HAART patients. Vitamin C concentrations were not different between Pre-HAART, Therapy II&III patients but patients on Therapy I had reduced levels of Vitamin C compare to pre-HAART (p < 0.01), therapy II &III patients (p < 0.001) (Table 5).

**Table 2:** The effect of HIV infection and the antiretroviral therapy, on markers of oxidative stress (carbonyls and TBARs).

**Groups**	**HIV negative (n = 90)**	**HIV positve**	
		**pre-HAART (n = 65)**	**HAART (n = 85)**
**Carbonyls (nmol/mg protein)**	1.07 ± 0.27	1.34 ± 0.98^a^	0.84 ± 0.90^a^
**TBARS (μmol/l)**	1.3 ± 0.12	4.2 ± 0.77^a^	6.28 ± 1.3^ab^

Significant differences (^a ^P < 0.0001) are by comparison with the control group (HIV Negative), (^b ^P < 0.0001) by comparison with the HIV positive patients without therapy (pre-HAART)

**Table 3:** The effect of HIV infection and the antiretroviral therapy, on plasma levels of antioxidants.

	**HIV negative (n = 90)**	**HIV**^+^	
**Groups**		**pre-HAART (n = 65)**	**HAART (n = 85)**
**Sulfhydryls (nmol/mg protein)**	8.03 ± 0.68	4.90 ± 2.78^a^	3.87 ± 2.85^a^
**Albumin (mol/l)**	0.94 ± 0.32	0.41 ± 0.05^a^	0.49 ± 0.28^ab^
**Vitamin C (μmol/l)**	27.1 ± 4.22	26 ± 4.01	21.43 ± 4.53

Significant differences (^a^P < 0.0001) are by comparison with the control group (HIV Negative), (^b^P < 0.0001) are by comparison with the HIV positive patients without therapy (pre-HAART)

**Table 4:** The effect of HIV infection and different types of antiretroviral therapy, on markers of oxidative stress (carbonyls and TBARs).

	**HIV^-^**	**HIV**^+^			
**Groups**	**Control (n = 90)**	**pre-HAART (n = 65)**	**Therapy I (n = 40)**	**Therapy II (n = 20)**	**Therapy III (n = 25)**

**Carbonyls (nmol/mg)**	1.07 ± 0.27	1.34 ± 0.98^a^	0.79 ± 0.10^ab^	0.93 ± 0.16^b^	0.81 ± 0.09^ab^
**TBARS (μmol/l)**	1.30 ± 0.12	4.20 ± 0.70^a^	7.02 ± 2.58^ab^	5.92 ± 1.46^ab^	5.84 ± 0.89^ab^

Significant differences (^a ^P < 0.01) are by comparison with the control group (HIV Negative), (^b ^P < 0.001) are by comparison with the HIV positive patients without therapy (pre-HAART)

**Table 5:** The effect different types of antiretroviral therapy, on plasma levels of antioxidants.

	**HIV^-^**	**HIV^+^**			
**Groups**	**Control (n = 90)**	**pre-HAART (n = 65)**	**Therapy I (n = 40)**	**Therapy II (n = 20)**	**Therapy III (n = 25)**

**Sulfhydryls (nmol/mg protein)**	8.03 ±± 0.68	4.90 ± 2.78^a^	4.5 ± 2.1^a^	2.70 ± 1.45^abc^	4.37 ± 2.01^a^
**Albumin (mol/l)**	0.94 ± 0.32	0.41 ± 0.05^a^	0.47 ± 0.24^a^	0.54 ± 0.31^ab^	0.47 ± 0.29^a^
**Vitamin C (μmol/l)**	27.1 ± 4.22	26 ± 4.01	12.9 ± 2.12^ab^	22.9 ± 5.01^c^	28.5 ± 7.45^c^

Significant differences (^a^P < 0.001) are by comparison with the control group (HIV Negative), (^b^P < 0.01) are by comparison with the HIV positive patients without therapy (pre-HAART) and (^c^P < 0.001) by comparison with the HIV positive patients on therapy I

**Table 6:** Comparison of oxidative stress markers levels between HIV infected patients with and without the CD4<200 lymphocytes/mm^3^

	**pre-HAART (n = 65)**		**HAART (n = 85)**	
**CD4 sub class**	<200	≥ 200	<200	≥ 200
**n**	28 (43,18%)	37 (56.82%)	44 (51.7%)	41(48.3%)
**BMI (kg/m^2^)**	23.91 ± 0.64	26.92 ± 1.09*	26.29 ± 1.39	25.22 ± 0.98
**Albumin (mol/l)**	0.39 ± 0.08	0.57 ± 0.13	0.50 ± 0.10	0.47 ± 0.15
**CD4 (lymphocytes/mm^3^)**	93.37 ± 13.01	434.21 ± 71.56**	73.26 ± 15.18	341.83 ± 30.44**
**Sulfhydryls (nmol/mg protein)**	4. 85 ± 1.30	4.94 ± 2.5	4.30 ± 2.7	3.59 ± 1.20
**TBARS (μmol/l)**	4.31 ± 0.47	4.82 ± 0.7	6.80 ± 1.62	5.82 ± 1.17
**Carbonyls (nmol/mg protein)**	1.45 ± 0.10	1.17 ± 0.16	0.83 ± 0.20	0.86 ± 0.14

Significant differences (**P < 0.005; **P < 0.0001) are by comparison with CD4 Sub class <200 lymphocytes/mm^3^

### Effect of nutritional status and CD4 count on oxidative damage seen in subjects on HAART

Our results showed a non significant difference in the oxidative stress markers levels between patients with and without CD4 count less than 200 lymphocytes/mm^3 ^(Table 7).

**Table 7:** The effect of weight status on Oxidative stress markers levels of HIV infected patients

	**pre-HAART (n = 65)**		**HAART (n = 85)**		
**BMI Sub class (kg/m^2^)**	20–25	≥ 25	<20	20–25	≥ 25
**n**	22 (33.9%)	43 (66.1%)	10 (11.8%)	42 (49.4%)	33 (38.8%)
**BMI (kg/m^2^)**	22.88 ± 0.48	27.63 ± 0.64	18.61 ± 0.32	23.04 ± 0.37	30.01 ± 0.94
**Albumin (mol/l)**	0.35 ± 0.03	0.51 ± 0.026^c^	0.57 ± 0.03	0.47 ± 0.027	0.40 ± 0.045
**Sulfhydryls (nmol/mg protein)**	5.02 ± 1.48	4.80 ± 2.12	3.60 ± 2.1	5.12 ± 2.5	3.39 ± 0.95^b^
**TBARS (μmol/l)**	4.09 ± 0.57	4.50 ± 0.77	5.57 ± 1.29	6.76 ± 1.50	6.40 ± 1.43
**Carbonyls (nmol/mg protein)**	0.93 ± 0.14	0.90 ± 0.2	1.09 ± 0.24	0.82 ± 0.15^a^	0.78 ± 0.11^a^

Significant differences (^a^P < 0.01) are by comparison with BMI Sub class <20 kg/m^2^

Significant differences (^b^P < 0.01;^c^P < 0.004) are by comparison with BMI Sub class 20–25 kg/m^2^

The effect of nutritional status was assessed using weight status criteria and plasma albumin level. Weight status was classified using body mass index, into the following categories: lean (<20 kg/m2), normal (20–24.9 kg/m2), overweight or obesity (>25 kg/m^2^). Among pre-HAART patients 33.9% had normal weight and 66.1% were overweight and only albumin level was significantly different between the two categories (Table 8). In HAART group, 11.8%, 49.4%, 38.8% patients were lean, normal or overweight respectively. In this group, protein oxidation was high in lean patients compare to normal or overweight patients. We noticed a significant decrease in antioxidant capacity (sulfhydryls group) in both lean and overweight patients.

**Table 8:** Comparison of oxidative stress markers levels between HIV infected patients with and without hypoalbuminemia

	**pre-HAART (n = 65)**		**HAART (n = 85)**	
**Albumin sub class (mol/l)**	<0.507	≥ 0.507	<0.507	≥ 0.507
**n**	35 (53.9%)	30 (25.9%)	63 (74.9%)	22 (25.9%)
**BMI (kg/m^2^)**	23.87 ± 0.65	27.83 ± 1.15**	25.11 ± 0.96	25.54 ± 1.47
**Albumin (mol/l)**	0.35 ± 0.094	0.57 ± 0.05***	0.33 ± 0.017	0.55 ± 0.018***
**CD4 (lymphocytes/mm**^3^**)**	345.71 ± 59.58	582.25 ± 102.49**	258 ± 42.04	269.38 ± 45.38
**Sulfhydryls (nmol/mg protein)**	4.73 ± 1.49	5.07 ± 2.7	3.52 ± 1.59	4.24 ± 2.71
**TBARS (μmol/l)**	3.95 ± 0.56	4.72 ± 0.64**	6.79 ± 1.21	6.11 ± 1.75*
**Carbonyls (nmol/mg protein)**	0.95 ± 0.17	0.81 ± 0.16	0.82 ± 0.16	0.85 ± 0.18

Significant differences (*P < 0.05; **P < 0.001; ***P < 0.0001) are by comparison with albumin Sub class <0.507 mol/l

A biochemical assessment of nutritional status using plasma albumin level cut off point of <3.5 g/dl (507 μmol/l) as indicator of malnutrition showed that 53.9% of pre-HAART and 74.1% of HAART patients had lower albumin level (Table 9). Lipid oxidation (TBARs) was increased in pre-HAART patients with normal albumin level and in HAART patients with lower albumin level. Protein oxidation process was not affected by albumin status (Table 8).

Multiple linear regression analysis confirmed that BMI sex, age and CD4 count and viral load did not had a significant impact on TBARs and carbonyls concentrations but CD4 and Viral load significantly influenced the antioxidant capacity (albumin and (sulfhydryls group) in HAART patients (Table 9).

**Table 9:** Multiple linear regression analysis showing the influence of BMI, age, sex, CD4 count and Viral load on oxidative stress markers in HAART patients

**Parameters**	**Independent contributing factors**				
	**BMI**	**Age**	**Gender**	**CD4**	**Viral Load**
**Carbonyls (nmol/mg protein**	0.093	0.140	0.026	-0.139	0.176
**Sulfhydryls (nmol/mg protein)**	0.104	-0.030	0.015	0.420**	-0.407**
**TBARS (μmol/l)**	0.170	0.060	0.185	-0.190	0.102
**Vitamin C (μmol/l)**	-0.067	-0.010	0.010	-0.091	-0.084
**Albumin (mol/l)**	0.345*	-0.011	0.030	0.320*	-0.366*

* p < 0.05, **p < 0.01.

## Discussion

The present study was designed to investigate the effect of antiretroviral therapies on oxidative stress markers in HIV infected patients in Cameroon. Voluntary screening for HIV infection is not common in Cameroon, and people will generally be diagnosed when they consult for illnesses and symptoms related to HIV infection. When patients are diagnosed as HIV positive, therapy is initiated when the CD4 count is less than 200 lymphocytes/mm^3^. The consequence of this is patients being on antiviral therapy when the HIV infection is fairly advanced. When therapy is however initiated, it varies depending on the therapeutic objectives, the cost, and their availability on the market. Our results showed that, HIV infection increases the oxidative stress process, which is further increased by the use of ART. This was observed by the significantly higher TBARs (lipid peroxidation) concentrations (Table 4), suggesting an increase in lipid peroxidation [10]. Among the three therapeutic regimes used patients on Therapy I which is the association of a commonly used fixed-dose combination therapy [11]^as ^monotherapy, thereby combining it with a generic fixed-dose combination of Lamivudin and zidovudin, showed lower plasma concentration of the antioxidant Vitamin C and higher TBARs. It is therefore possible that, the observed effect may be a consequence of this association. Others studies reported that HAART medications may increase oxidative stress levels above and beyond levels caused by the virus itself. In HIV infection, reactive oxygen species may enhance viral replication by activating nuclear transcription factors, which ultimately lead to viral gene expression. Further, in HIV-infected adults, zidovudin was shown to promote oxidative damage to DNA, a process that was reversible with vitamin C and vitamin E supplementation [12]. Several pre-HAART studies found that both asymptomatic HIV-infected individuals and AIDS patients had higher levels of oxidative stress, as indicated by increased plasma metabolites of lipid peroxidation and/or reduced antioxidant levels, compared with healthy controls [13,14]. HAART may induce (i) an increase in oxidant generation, (ii) a decrease in anti-oxidant protection, or (iii) a failure to repair oxidative damage. Oxidative stress-mediated cell damage occurs, in part, via reactive oxygen species (ROS). ROS include molecules like hydrogen peroxide; ions like the hypochlorite ion; radicals like the hydroxyl radical; and the super oxide anion which is both ion and radical. Radicals (also called "free radicals") are a cluster of atoms that contain an unpaired electron in their outermost orbit of electrons. This is an extremely unstable configuration, and radicals quickly react with other molecules or radicals to achieve the stable configuration. Once formed, ROS participate in a number of reactions, yielding additional free radicals such as hydrogen peroxide, peroxynitrite, or hypochlorous acid [15]. The free radicals in HIV infection could result also from non-enzymatic protein oxidation and the subsequent oxidative degradation of glycated proteins.

We noticed that, increase in oxidative stress brought about by HIV infection as well as the use of therapy was paralleled by significant decreases in glutathione (GSH) level (sulfhydryls group) and albumin, as well as Vitamin C with Therapy I. Abnormally high levels of free radicals as well as the simultaneous decline of antioxidant defence mechanisms can lead to damage of cellular organelles and enzymes as well as increased lipid peroxidation. Under normal conditions, numerous cellular antioxidant systems exist to defend against oxidant stress and maintain the redox balance of the cell. ROS are cleared from the cell by enzymatic systems including superoxide dismutases (SODs), catalase, and glutathione peroxidase, or the nonenzymatic system including alpha-tocopherol (vitamin E), ascorbic acid (vitamin C), glutathione, and uric acid. Glutathione peroxidase plays an important role as defense mechanism in mammals, against oxidative damage by catalyzing the reduction of a variety of hydroperoxides, using glutathione as the reducing substrate. In addition to its role as a substrate in GSH redox cycle, glutathione, also act as a direct endogenous scavenger of hydroxyl radicals, involved in detoxification and metabolism of a number of substances in the liver [16]. HAART may reduce GSH synthesis, enhanced GSH utilization, or limited intracellular reduction of its oxidized form (GSSG) [16]. As a consequence of GSH deficiency, a number of related functions may be impaired such as a decrease in reducing capacity, protein biosynthesis, immune function, accumulations of lipid peroxidation products and detoxification capacity. A reduced detoxification capacity in the liver may lead to the accumulation of hepatotoxic metabolites leading to liver damage [16,17]. Antiviral therapy could also have a role in oxidative stress resulting from the destruction of tissues and liver cells and the activation of neutrophils and macrophages. The major damage to cells results from the ROS-induced alteration of macromolecules such as polyunsaturated fatty acids in membrane lipids, essential proteins and DNA [18]. Indirect oxidative damage of proteins is also possible, through their interaction with reactive carbonyl compounds formed by the auto-oxidation of carbohydrates and lipids [19,20]. The differences in oxidative stress as well as in the plasma concentrations of antioxidants between pre HAART patient and HAART patient may be explain by their nutritional status. Malnutrition can have a severe impact on the specific antigen-antibody components of the immune system and can also compromise general bodily defence mechanisms [20] and was common in HIV-infection prior to the introduction of highly active antiretroviral therapy (HAART). Although HAART result in suppression of viral replication and dramatic improvement in clinical, immunological [21], and nutritional status, as shown our results, weight loss and wasting may still be observed in some patients on ARV treatment. We noticed that, 10 % of HAART patients were underweight and chronic diarrhoea, opportunist infections, were the primary contributors to these nutritional deficiencies and may induce a decrease in the level of antioxidant capacity (sulfhydryls group). The increase in lipid peroxidation found in HAART patients may also be explaining by the lipodystrophy syndrome. Previous studies suggested that lipodystrophy syndrome in HIV-positive individuals on HAART is characterized by subcutaneous fat wasting, visceral fat accumulation, lipid abnormalities, and insulin resistance or glucose intolerance [22]. Certain aspects of this syndrome may be associated with oxidative stress, thereby increasing the body's demand for certain antioxidants [23,24]

## Conclusion

HIV infection increases the oxidative stress process, while anti retroviral combination therapy compounds this effect by increasing lipid oxidation. The decrease in antioxidants that accompanies HIV infection suggests a potentially important role of nutritional supplementation and good nutrition in general in the proper management of HIV/AIDS.
